# A survey of the treatment and management of ingrown toenails by UK podiatrists: A cross‐sectional survey

**DOI:** 10.1002/jfa2.12017

**Published:** 2024-06-04

**Authors:** Victoria Exley, Katherine Jones, Judith Watson, Michael Backhouse

**Affiliations:** ^1^ Department of Health Sciences York Trials Unit University of York York UK; ^2^ Warwick Clinical Trials Unit Warwick Medical School University of Warwick Coventry UK

**Keywords:** ingrown nails, malformed, nail avulsion, nail surgery, onychocryptosis, questionnaires, survey

## Abstract

**Background:**

Ingrown toenails are a common pathology. Although a range of conservative and surgical measures are widely used for this condition, little is known about their use in practice. This study explored current practice relating to the treatment or management of ingrown toenails by podiatrists in the UK.

**Methods:**

A cross‐sectional online survey (Qualtrics, Provo, UT, USA) conducted between March to June 2020 was distributed to practicing podiatrists treating or managing ingrown toenails in the UK.

**Results:**

A total of 396 practicing podiatrists responded (60.1% based in the private sector). The majority (88.6%) performed nail surgery most commonly (54.3%) less than five a month. Nearly all (95%) only performed nail avulsion with or without chemical matrixectomy, universally using phenol (97.2%). Application time and number of applications varied but was most commonly applied three times (61.5%) for a total of 3 minutes (75%). Aftercare varied considerably between public and private sectors, with public sectors offering fewer follow‐up appointments.

**Conclusions:**

Although there is a variation in clinical practice throughout the treatment pathway, almost all respondents offered nail avulsion with phenol matrixectomy, whereas very few provided incisional nail surgery. This data provides the most comprehensive description of how UK podiatrists conduct nail surgery for onychocryptosis.

**Abbreviations**
CROSS: Consensus‐based checklist for Reporting of Survey Studies reporting guideline
NHS: National Health Service
PROMs: Patient Reported Outcome Measures
UK: United Kingdom

## 1 INTRODUCTION

An ingrown toenail, or onychocryptosis, is a common pathology that occurs when the nail plate punctures the periungual skin, often causing pain, inflammation and infection if left untreated [1]. Whilst mild, early, cases can be treated with conservative measures, many require surgical care to relieve symptoms. Indeed, it is so common, that nail surgery has been identified as the tenth most common procedure performed by UK podiatrists [2].

Conservative approaches include appropriate nail cutting and spicule removal, packing the nail sulcus, and orthonyxia (nail bracing), which have all been advocated for use in mild to moderate (stage I and stage II) onychocryptosis [3, 4]. When conservative treatment fails, where there is nail deformity, or in more severe cases (stage II and III), a surgical approach is often recommended [5]. Although there are multiple procedures and options for performing such surgery, they typically aim to remove the problematic part of the nail and destroy the nail matrix to avoid painful regrowth [4, 5, 6].

A recent systematic review and meta‐analyses concluded that despite the high number of publications on nail surgery, very few conclusions could be drawn from the evidence due to the poor quality of research [7, 8]. Notably when studies were assessed using the Cochrane RoB 2.0 tool, all studies were graded as having an overall bias of either ‘some concerns’ or ‘high risk’. With such a limited evidence base, there is little to guide clinician's practice. Although in the UK, the Royal College of Podiatry offer clinical practice guidelines that are not prescriptive in nature and formally auditing against them may be challenging [5]. An alternative approach for clinicians seeking to evaluate the quality of their service and seek improvements is benchmarking, which is increasingly used across many different sectors including healthcare [9, 10].

Although there are numerous definitions of the term, and it can be seen as a structured methodology in quality improvement [11], at its heart, benchmarking is a process of peer comparison, which has been shown to detect and reduce unwarranted variation [10, 12, 13]. To enable such comparative benchmarking in nail surgery, it is first necessary to describe current practice, but we were unable to find any contemporary data describing UK practice. Therefore, we aimed to describe current practice relating to the treatment of ingrown toenails by podiatrists in the UK.

## 2 METHODS

### 2.1 Study design

A cross‐sectional online survey was conducted between March 23, 2020 to June 4, 2020. Findings are reported in accordance with the Consensus‐based checklist for Reporting of Survey Studies (CROSS) reporting guideline [14].

### 2.2 Data collection methods

Survey questions were drafted by experienced clinicians and foot and ankle researchers with the aim of questions to explore clinical practice. The questionnaire was circulated to 12 podiatrists, of which 7 provided comments and piloted the questionnaire before changes were made and the final survey was circulated. The final survey comprised 54 questions; the first section collected questions about participants' practice, location and sector and highest level of qualification. The second section explored the number of ingrown toenails treated, use of classification systems, treatments offered and types of conservative treatments offered. The third section focussed on the surgical treatment of ingrown toenails including pre, peri and post procedural details, the latter focussing on aftercare, patient outcomes and audits (Supplementary File 1).

### 2.3 Sample characteristics

Participants were UK based and required to be currently practicing podiatry treating and/or managing ingrown toenails, regardless of whether they worked in the public or private sector. Therefore, any participants not based in the UK or not working with ingrown toenails were excluded.

### 2.4 Survey administration

We used a cross‐sectional, self‐administered, anonymous survey to elicit the details of clinical practice using the Qualtrics online survey platform (Qualtrics, Provo, UT, USA). It was circulated by email to all members of the Royal College of Podiatry and advertised on social media including Twitter, the UK Podiatry, UK Podiatry Business and Podiatry UK Facebook groups. Consent was implied by survey completion.

### 2.5 Statistical analysis

All responses collected through Qualtrics were inspected and downloaded to Excel, coded and analyzed using descriptive statistics (frequencies and percentages). We made an a priori decision to present public and private sector data separately but not to conduct inferential statistics due to the large number of variables collected and the lack of existing data upon which to base hypotheses or sample size calculations. All returned surveys were included in the analysis regardless of the level of completion; therefore, the total number of responses for each response vary due to missing data.

## 3 RESULTS

### 3.1 Participant and professional characteristics

Overall, there were 396 eligible respondents to the survey of whom 297 (75%) were based in England (Table 1). One hundred and twenty‐two (30.8%) respondents were in practice for 0–10 years, and the majority described working primarily in the private sector (*n* = 238/396; 60.1%). Undergraduate degrees were the most common reported level of education (*n* = 265/396; 66.9%).

**TABLE 1:** Participant and professional characteristics.

	Private sector [*n* = 238]	Public sector [*n* = 158]	All [*n* = 396]
Location based, *n* (%)			
England	189 (79.4)	108 (68.4)	297 (75.0)
Northern Ireland	6 (2.5)	9 (5.7)	15 (3.8)
Scotland	24 (10.1)	31 (19.6)	55 (13.9)
Wales	19 (8.0)	10 (6.3)	29 (7.3)
Years in practice, *n* (%)			
0–10 years	85 (35.7)	37 (23.4)	122 (30.8)
11–20 years	59 (24.8)	50 (31.6)	109 (27.5)
21–30 years	66 (27.7)	42 (26.6)	108 (27.3)
31–40 years	21 (8.8)	26 (16.5)	47 (11.9)
41+ years	6 (2.5)	3 (1.9)	9 (2.3)
Highest education, *n* (%)			
Diploma	33 (13.9)	13 (8.2)	46 (11.6)
Undergraduate Degree	169 (71.0)	96 (60.8)	265 (66.9)
Master's Degree	36 (15.1)	41 (25.9)	77 (19.4)
PhD	0 (0.0)	4 (2.5)	4 (1.0)
Professional Doctorate	0 (0.0)	4 (2.5)	4 (1.0)

Abbreviation: *n*, number.

### 3.2 Clinical characteristics

**FIGURE 1:**
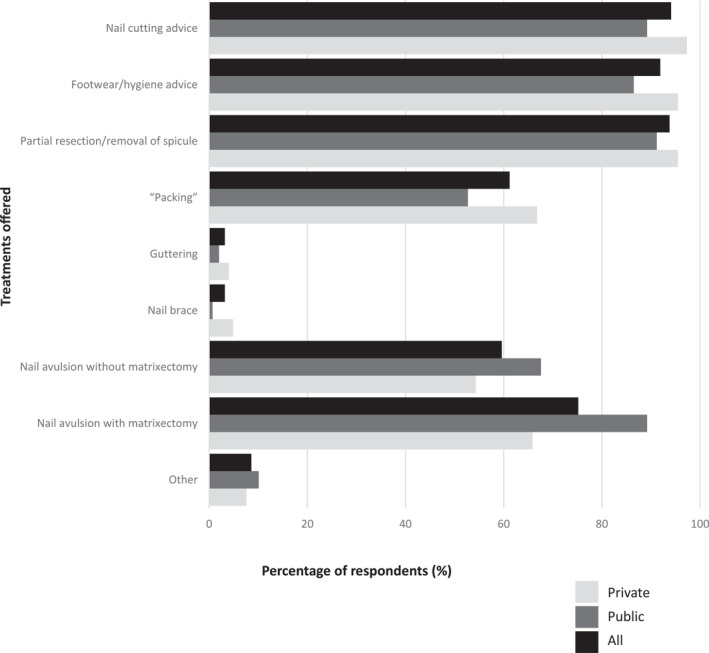
Treatment offered (*n* = 371; private *n* = 223, public *n* = 148). *Multiple answers possible.

Almost 72% of respondents treated five or more ingrown toenails per month, although over 80% did not use a classification or grading system to quantify severity (*n* = 325/389; 83.5%) (Table 2). For those who utilised a classification or grading system, more than three‐quarters used their own system (*n* = 40/51; 78.4%). Figure 1 shows the type of treatments offered, the most common being nail cutting advice (94.1%), partial resection (93.8%) and footwear/hygiene advice (91.9%). It was notable that packing was offered less in the public sector than the private sector (52.7% vs. 66.8%), whereas nail avulsion with or without matrixectomy was more commonly offered in the public sector. Supplementary Figure S1 shows that bracing systems are not commonly used in the public or private sector.

**TABLE 2:** Clinical characteristics.

	Private sector [*n* = 233]	Public sector [*n* = 156]	All [*n* = 389]
Number treated per month, *n* (%)			
<5	82 (35.2)	28 (17.9)	110 (28.3)
5–10	74 (31.8)	39 (25.0)	113 (29.0)
11–20	47 (20.2)	51 (32.7)	98 (25.2)
21+	30 (12.9)	38 (24.4)	68 (17.5)
Grading/Classification system, *n* (%)			
No	197 (84.5)	128 (82.1)	325 (83.5)
Yes	36 (15.5)	28 (17.9)	64 (16.5)
*If yes* ^a^: Own system	23 (79.3)	17 (77.3)	40 (78.4)
Mozena [15]	3 (10.3)	1 (4.5)	4 (7.8)
Zuber [16]	1 (3.4)	0 (0.0)	1 (2.0)
Other	2 (7.0)	4 (18.2)	6 (11.8)

Abbreviation: *n*, number.

^a^ 20% (*n* = 13/64) of respondents did not complete this question.

### 3.3 Surgical characteristics

**FIGURE 2:**
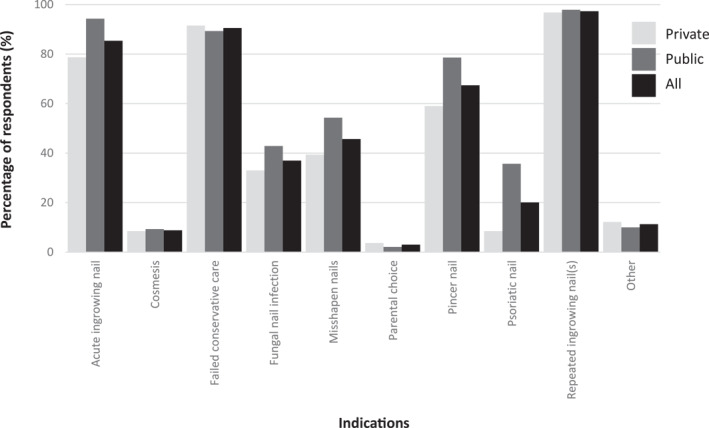
Most common indication for performing nail surgery (*n* = 328; private *n* = 188, public *n* = 140)*. *Multiple answers possible.

Most respondents perform nail surgery (*n* = 326/370; 88.6%), most commonly less than five nail surgeries per month (*n* = 201/328; 54.3%); however, more surgeries (11+) were notably reported by in the public sector (37.9% vs. 2.6%). Nearly all respondents (*n* = 310/328; 94.5%) reported that they only perform nail avulsion with/without chemical matrixectomy, with nearly all routinely including chemical matrixectomy (*n* = 254/270; 94.1%). The surgical team varied between public and private sectors, with more surgeries being carried out alone in the private sector than public (32.9% vs. 9.7%) and more surgeries being carried out with an assistant in the public sector than private (74.3% vs. 41.1%). Over half of the respondents refer patients to a surgeon for other types of nail surgery. This was largely due to medical considerations (73.7%) or when the patient preferred general anesthetic over local anesthetic (63.7%). For those that do perform other procedures, these included Zadiks, Winograd, Frost and Suppan. The most common reason for not performing surgery for those working in the private sector was working alone and for those in the public sector it was not part of the current job role (Table 3). The most common indication for performing nail surgery was repeated ingrowing nail(s) (97.3%), failed conservative care (90.5%) and acute ingrowing nails (85.4%) (Figure 2).

**TABLE 3:** Surgical characteristics.

	Private sector [*n* = 222]	Public sector [*n* = 148]	All [*n* = 370]
Nail surgery performed, *n* (%)			
Yes	189 (85.1)	137 (92.6)	326 (88.6)
No	33 (14.9)	11 (7.4)	44 (11.4)
*If no* ^a^ ^,^ ^g^ *, why:* Not part of the current role	3 (11.1)	9 (90.0)	12 (30.8)
Work alone	20 (74.1)	0 (0.0)	20 (51.3)
Not enough demand	6 (22.2)	0 (0.0)	6 (15.4)
Fear of litigation	3 (11.1)	0 (0.0)	3 (7.7)
Other	5 (18.5)	1 (10.0)	6 (15.4)
Number of nail surgeries performed per month, *n* (%)^b^			
<5	159 (84.6)	42 (30.0)	201 (54.3)
5–10	24 (12.8)	45 (32.1)	69 (18.6)
11–20	4 (2.1)	26 (18.6)	30 (8.1)
21+	1 (0.5)	27 (19.3)	28 (7.6)
Type of nail surgery performed, *n* (%)^b^ ^,^ ^g^			
Only nail avulsion (with/without chemical matrixectomy)	182 (96.8)	128 (91.4)	310 (94.5)
Suppans procedure	0 (0.0)	2 (1.4)	2 (0.6)
Zadiks procedure	4 (2.1)	8 (5.7)	12 (3.7)
Frost procedure	3 (1.6)	5 (3.6)	8 (2.4)
Winograd procedure	4 (2.1)	12 (8.6)	16 (4.9)
Other	3 (1.6)	3 (2.1)	6 (1.8)
Use chemical matrixectomy, *n* (%)^c^			
Yes	142 (91.6)	112 (97.4)	254 (94.1)
No	13 (8.4)	3 (2.6)	16 (5.9)
Surgical referral for other types of nail surgery, *n* (%)^d^			
Yes	109 (58.0)	91 (61.5)	200 (59.5)
No	77 (41.0)	51 (34.5)	128 (38.1)
Respondent was a surgeon	2 (1.0)	6 (4.0)	8 (2.4)
If yes, reasons for referral to surgeon, *n* (%)^e^ ^,^ ^g^			
Medical considerations	71 (75.5)	61 (71.8)	132 (73.7)
Failed chemical matrixectomy	29 (30.9)	15 (17.6)	44 (24.6)
General anesthetic preferred by patient	56 (59.6)	58 (68.2)	114 (63.7)
Other	24 (25.5)	4 (4.7)	28 (15.6)
Surgical team, *n* (%)^f^ ^,^ ^g^			
Alone	52 (32.9)	11 (9.7)	63 (23.3)
With another podiatrist	56 (35.4)	43 (38.1)	99 (36.5)
With an assistant	65 (41.1)	84 (74.3)	149 (55.0)
Other	18 (11.4)	9 (8.0)	27 (10.0)

Abbreviation: *n*, number.

^a^ 11.4% (*n* = 5/44) of respondents did not complete this question.

^b^ 11.4% (*n* = 42/370) of respondents did not complete this question.

^c^ 27.0% (*n* = 100/370) of respondents did not complete this question.

^d^ 9.2% (*n* = 34/370) of respondents did not complete this question.

^e^ 10.7% (*n* = 21/200) of respondents did not complete this question.

^f^ 26.8% (*n* = 99/370) of respondents did not complete this question.

^g^ Multiple answers possible.

#### 3.3.1 Pre‐procedure

**FIGURE 3:**
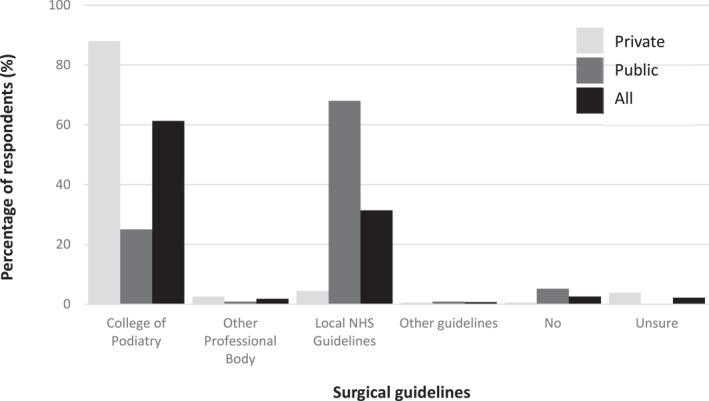
Use of surgical guidelines (*n* = 274; private *n* = 158, public *n* = 116).

Nearly all respondents (*n* = 267/274; 97.4%) reported using surgical guidelines, most commonly the Royal College of Podiatry for those in the private sector (*n* = 139/158; 88%) and unsurprisingly the local NHS guidelines for those in the public sector (*n* = 79/116; 68%) (Figure 3). Prior to nail surgery, most respondents ‘sometimes’ provided a course of antibiotics if local infection was present (*n* = 173/328; 52.8%) and performed vascular (*n* = 272/274; 99.3%) and neurological (*n* = 217/274; 79.2%) pre‐procedure checks (Supplementary Table S1 and Supplementary Figure S2). Supplementary Table S2 illustrates the pre‐surgical management of health conditions and medication.

#### 3.3.2 Procedure

Most respondents used a tourniquet (99.3%), did not mask the surrounding skin (*n* = 157/244; 64.3%), either did (*n* = 60/244; 24.6%) or “sometimes” (*n* = 112/244; 45.9%) used a sharp dissection to remove hypergranulation tissue. There was a variation in whether the area was irrigated or flushed, with over a third advising they use chlorhexidine (*n* = 84/244; 34.4%) or do not irrigate/flush (*n* = 81/244; 33.2%). Mepivacaine and phenol [Liquid 58.6% or EZ Swabs® 38.1%] were the most common local anesthetic and chemical used (Table 4).

**TABLE 4:** Surgical characteristics: Procedure.

	Private sector [*n* = 155]	Public sector [*n* = 115]	All [*n* = 270]
Use of tourniquet, *n* (%)			
Yes	154 (99.3)	114 (99.1)	2698 (99.3)
No	1 (0.7)	1 (0.9)	2 (0.7)
Use a protect mask the surrounding skin, *n* (%)			
Yes	53 (39.0)	34 (31.5)	87 (35.7)
No	83 (61.0)	74 (68.5)	157 (64.3)
Sharp dissection to remove hypergranulation tissue, *n* (%)^a^			
Yes	29 (21.3)	31 (28.7)	60 (24.6)
Sometimes	65 (47.8)	47 (43.5)	112 (45.9)
No	42 (30.9)	30 (27.8)	72 (29.5)
Irrigate/flush used^a^			
Chlorhexidine	48 (35.3)	36 (33.3)	84 (34.4)
Clinisept®	6 (4.4)	2 (1.9)	8 (3.3)
Saline solution	28 (20.6)	16 (14.8)	44 (18.0)
Other	13 (9.6)	14 (13.0)	27 (11.1)
Do not irrigate/flush	41 (30.1)	40 (37.0)	81 (33.2)
Local anesthetic used, *n* (%)^b^			
Lidocaine	21 (13.5)	23 (20.0)	44 (18.0)
Mepivacaine	130 (83.9)	104 (90.4)	234 (86.7)
Prilocaine	8 (5.2)	4 (3.5)	12 (4.4)
Bupivocaine	5 (3.2)	8 (6.9)	13 (4.8)
Levobupivocaine	0 (0.0)	11 (9.6)	11 (4.0)
Ropivocaine	0 (0.0)	5 (4.3)	5 (1.9)
Lidocaine with adrenaline	1 (0.6)	2 (1.7)	3 (1.1)
Other	0 (0.6)	2 (1.7)	2 (0.7)

Abbreviation: *n*, number.

^a^ 9.6% (*n* = 26/270) of respondents did not complete this question.

^b^ Multiple answers possible.

**FIGURE 4:**
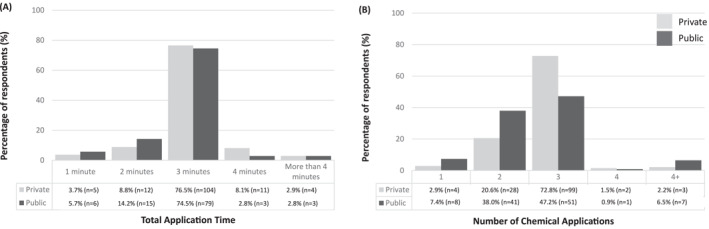
Total application time (A) and number of chemical applications offered (B) (*n* = 244).

Application time and number of applications varied, but most commonly was applied three times (*n* = 150/244; 61.5%) for a total of 3 minutes (*n* = 183/244; 75%) (Figure 4). Notably, Iodine (*n* = 70/155; 45.2%) was the most commonly used disinfectant in the private sector as opposed to Chlorhexidine (*n* = 59/115; 51.3%) in the public sector (Supplementary Figure S3). Primary, secondary and tertiary dressings applied can be found in Supplementary Table S3. Products provided for aftercare varied, with private sectors providing more dressings, tubegauz (or equivalent), hypafix®/tape, saline solution and clinisept® than the public sector (Supplementary Table S1).

#### 3.3.3 Follow‐up

Care pathways varied between private and public sectors, with public sectors offering fewer follow‐up appointments following total and partial nail avulsion, and more commonly appointments were only offered if complications were present (Table 5). Healing time for both total and partial nail avulsion were similar, with both sectors estimating 4‐–9 and 4–6 weeks, respectively, and advising on saltwater bathing between dressing changes (63.5%). Interestingly, only half (*n* = 134; 51.5%) of the respondents evaluate the outcomes of nail surgery, slightly more common in the public sector than in the private sector (56.8% vs. 47.7%). These evaluations most often included patient satisfaction (92%), were completed within 1–6 months (59.2%) and were most commonly undertaken via a telephone consultation in the public sector (44.1%) or via a follow‐up appointment (60.6%) in the private sector. Lastly, more than half of the respondents (*n* = 75/125; 60%) informed that they did not or were unsure whether they used a professional standard/guideline to audit compliance (Supplementary Table S1).

**TABLE 5:** Surgical follow‐up.

	Private sector [*n* = 150]	Public sector [*n* = 110]	All [*n* = 260]
Follow‐up appointments for total nail avulsion, *n* (%)			
1–3	101 (67.3)	81 (73.6)	182 (70)
4–6	37 (24.7)	8 (7.3)	45 (17.3)
7–9	5 (3.3)	1 (0.9)	6 (2.3)
10+	5 (3.3)	0 (0.0)	5 (1.9)
None	2 (1.3)	20 (18.2)	22 (8.5)
Follow‐up appointments for partial nail avulsion, *n* (%)			
1–3	111 (74.0)	82 (74.5)	194 (74.6)
4–6	31 (20.7)	6 (5.5)	37 (14.2)
7–9	2 (1.3)	0 (0.0)	2 (0.8)
10+	3 (2.0)	1 (0.9)	4 (1.5)
None	3 (2.0)	20 (18.1)	23 (8.8)
Appointment wait after surgery^a^			
1–3 days	107 (71.8)	69 (62.7)	176 (67.9)
3–7 days	36 (24.2)	17 (15.5)	53 (20.5)
7+ days	4 (2.7)	8 (7.3)	12 (4.6)
Only if complications are present	2 (1.3)	16 (14.5)	18 (6.9)

Abbreviation: *n*, number.

^a^ 0.4% (*n* = 1/260) of respondent did not complete this question.

## 4 DISCUSSION

This study aimed to describe UK practice around nail surgery in order to enable clinicians to benchmark their service due to the lack of existing data. Our online survey achieved 396 eligible responses in a short time window. With 72% conducting five or more and 17.5% conducting over 21 procedures per month, we have confirmed how commonly UK podiatrists perform nail surgery.

Although numerous surgical procedures have been described to treat ingrown toenails, 95% of respondents only performed nail avulsion with or without chemical matrixectomy rather than incisional nail surgery. Of the incisional nail procedures respondents offered, Winograd (4.9%) was the most common, followed by Zadiks (3.7%). Similarly, phenol was almost universally used for chemical matrixectomy with 97.2% of respondents using it. With such a large proportion only providing one procedure or one chemical, it is perhaps surprising that a recent systematic review identified that the current evidence base does not demonstrate superiority of one procedure or chemical over others for outcomes of relief of symptoms, symptomatic regrowth, healing time, post‐operative complications, pain or patient satisfaction [7, 8]. Similarly, with Royal College of Podiatry guidelines stating that surgical excision may be considered where healing capacity is reduced, and primary intention would be more suitable, it is clear that most podiatrists would need to refer patients to enable this [17].

Phenol is a volatile organic acid used to destroy tissue in the nail matrix and prevent the pathological nail from re‐growing. Clinicians must determine how long to apply the phenol in order to maximise the likelihood of a successful procedure whilst minimising the chances of excessive, unnecessary tissue damage. Our data showed that practice in the application of phenol varied between respondents and sectors. Although Royal College of Podiatry guidelines suggest three, one‐minute applications of phenol in a well perfused hallux and this was the most frequently reported application pattern, our data shows that a range of timings are used in practice, with many using two applications. It is also notable that a larger proportion of respondents working in the public sector reported typically using two applications when compared to the private sector. Although it is important to clarify that this variation is not precluded within the guidelines, it does perhaps reflect the lack of evidence to inform decision making on key elements of the procedure [5, 7, 8].

Matrixectomy is considered a definitive treatment as it is used to prevent nail regrowth, but in our survey 59.6% of respondents said they offered nail surgery without matrixectomy. That is nail surgery to remove part or all of the effected nail, but enable it to regrow. This approach is advocated in the literature for a broad spectrum of indications including: when there is a singular incidence of ingrown toenail with no underlying nail pathology; removal of fungal nail in preparation for treatment; and poor healing capacity [18]. In addition to specific clinical indications for this procedure, there is also increasing awareness of the post‐operative cosmetic appearance following nail surgery [19]. Given the breadth of indications for nail avulsion without matrixectomy, it seems surprising that 40% do not offer this option and suggests that matrixectomy may be over‐used in practice and there may be opportunities to improve the use of shared decision making in this process.

The variation seen between the public and private sector in terms of aftercare is perhaps not surprising. Although the most common response in both sectors was 1–3 appointments, those in the private sector appear more likely to offer 4–6 follow‐up appointments (24.7% vs. 7.3%), and those in the public sector were more likely to say that they did not routinely offer any follow‐up appointments (1.3% vs. 18.2%). Even when just looking at follow‐up within the public sector, this data suggests considerable variation with 18.2% not offering any routine follow‐up appointments, and 73.6% offering 1–3 appointments. Reducing the number of follow‐up appointments and moving toward patient initiated follow‐up is likely to provide considerable cost saving for services if it can be confirmed as a safe and effective model of care.

The relatively low proportion of respondents reporting that they assessed the outcome of their nail surgery was also noteworthy. Whilst exploring reasons behind this omission was beyond the scope of the current survey, it does warrant consideration in future research. Use of Patient Reported Outcome Measures (PROMs) is now widespread in healthcare but to our knowledge, there is an absence of validated PROMs for use in nail surgery, no core outcome set and very limited literature around what matters to patients. Even prior to this work, clinical data can be audited to monitor ‘hard outcomes’, such as infection rates, healing times and rates of regrowth.

The primary limitation of the data presented here is that they were collected in 2020 and it is possible that practice has changed since then, particularly in the delivery of chemical matrixectomy following a National Patient Safety Announcement on the use of bottled liquid phenol in 2021 [20]. Following this, the Royal College of Podiatry amended their guidelines and now recommend that only UKCA labeled phenol application products should be used [17]. Whilst this is likely to change the technique used to apply phenol during matrixectomy, it is not clear whether this has led to a change in the chemical used to destroy the nail matrix. Despite this, there has not been any similar data presented on national practice of nail surgery in the interim and this remains the most recent UK data to enable benchmarking.

The survey was open between March and June 2020, and this coincides with the first wave of the COVID‐19 pandemic in the UK, which may have affected response rates. During this period, podiatrists were instructed by the UK government to only provide urgent care, which affected many private podiatrists, and a number of NHS podiatrists were redeployed to other areas [21]. Conversely, it is also possible that we would not have had such a large response to such a detailed questionnaire had we asked outside this window, as usual working patterns for a lot of podiatrists were altered during this time.

As has been stated in a recent systematic review, there is a lack of research into core aspects of the surgical management of ingrown toenails [7, 8]. Building on results from this survey, future research should seek to confirm the safety and effectiveness of reduced patient follow‐up and even patient initiated follow‐up. There also appears a need to explore the use of shared decision making around the use of matrixectomy, and to explore benefits of excisional nail surgery over chemical matrixectomy. Finally, future research should seek to repeat this survey in order to explore changes in practice over time and enable continued benchmarking of a core aspect of podiatric practice.

## 5 CONCLUSIONS

Our survey achieved a high number of responses and confirms how commonly UK podiatrists perform nail surgery. Although there is variation in clinical practice throughout the treatment pathway, almost all respondents offered nail avulsion with phenol matrixectomy, whereas very few provided incisional nail surgery. This data provides the most comprehensive description of how UK podiatrists conduct nail surgery for onychocryptosis. For the first time this will enable clinicians to benchmark their practice against their peers throughout the UK.

## AUTHOR CONTRIBUTIONS

**Victoria Exley**: Conceptualisation; methodology; project administration; data curation; formal analysis; writing – original draft; writing – review & editing. **Katherine Jones**: Methodology; data curation; formal analysis; writing – original draft; writing – review & editing. **Judith Watson**: Conceptualisation; methodology; supervision; data curation; formal analysis; writing – original draft; writing – review & editing. **Michael Backhouse**: Conceptualisation; methodology; supervision; data curation; formal analysis; writing – original draft; writing – review & editing.

## CONFLICT OF INTEREST STATEMENT

The authors declare that they have no competing interests.

## ETHICS STATEMENT

The study was approved by the University of York Department of Health Sciences Research Ethics Committee in February 2020.

## Supporting information

Supporting Information S1

## Data Availability

The datasets analyzed during the current study are available from the corresponding author on reasonable request.
