# Supplementary material for: A survey of the treatment and management of ingrown toenails by UK podiatrists: A cross‐sectional survey
Source: J Foot Ankle Res. 2024 Jun 4;17(2):e12017. doi: 10.1002/jfa2.12017 (PMC11296716; doi:10.1002/jfa2.12017)
Supplement: Supplementary file 1 — Supporting Information S1 [file JFA2-17-e12017-s001.docx]

**Supplementary Material Content**

**Supplementary File 1.** Qualtrics Survey

**Supplementary Figure 1.** Packing and nail bracing offered

**Supplementary Table 1.** Surgical characteristics

**Supplementary Figure 2.** Vascular and Neurological Checks

**Supplementary Table 2.** Management of patients with certain health conditions and taking certain medications

**Supplementary Figure 3.** Disinfectants used

**Supplementary Table 3.** Dressings applied immediately after procedure

**Supplementary File 1**

Ingrowing Toenail Questionnaire

Start of Block: Informed Consent

Q1

Welcome to the research study!  


We would like to invite you to take part in the above research project which is being conducted as part of a MSc in Applied Health Research at The University of York. 


The study aims to evaluate the evidence available for the treatment and management of ingrowing toenails and describe current clinical practice amongst podiatrists. Anecdotally, practice varies greatly within the UK and internationally but there are no data describing this yet, and to date there is no consensus on best practice. This study aims to determine current practice amongst UK podiatrists.


You have been approached to participate as you are currently a practicing podiatrist. This survey is anonymous and neither I nor my academic supervisors have seen or have access to any of your contact details.


The research is entirely voluntary. The study involves a short, online survey regarding your current clinical practice when treating ingrown toenails. The survey should take approximately 10-15 minutes to complete.


You can withdraw from the study at any point, however as this survey is anonymous we are unable to withdraw your data once it has been entered into the survey. Any data provided up to the point of withdrawal will still be used. 


This study has been reviewed and approved by the Health Sciences Research Governance Committee at the University of York.


Thank you for taking the time to read this information sheet. Please note that this survey will be best displayed on a laptop or desktop computer.  Some features may be less compatible for use on a mobile device. 

End of Block: Informed Consent

Start of Block: About you

Q2 Where are you based?

- England (1)
- Northern Ireland (2)
- Scotland (3)
- Wales (4)
- Other-please specify (5) __________________________________________________

Q3 How many years have you worked in practice?

▼ 0-10 years (1) ... 40 years + (5)

Q4 What is your highest level of qualification?

- Diploma (1)
- Undergraduate degree (2)
- Masters degree (3)
- PhD (4)
- Professional doctorate (5)

Q5 Do you work primarily in the public or private sector? (if both please answer in respect of your primary role)

- Private (1)
- Public (2)

End of Block: About you

Start of Block: Treatment of ingrowing toenails

Q6 Do you treat people with ingrowing nails?

- Yes (1)
- No (If no, you will be taken to the end of the survey. Thank you for your time) (2)

Skip To: End of Survey If Do you treat people with ingrowing nails? = No (If no, you will be taken to the end of the survey. Thank you for your time)

Q7 How many ingrowing toenails do you treat in an average working month?

- <5 (1)
- 5-10 (2)
- 11-20 (3)
- 21+ (4)

Q8 Do you use a grading/classification system to quantify the severity of ingrowing toenails?

- Yes (1)
- No (2)

Skip To: Q9 If Do you use a grading/classification system to quantify the severity of ingrowing toenails? = Yes

Skip To: Q10 If Do you use a grading/classification system to quantify the severity of ingrowing toenails? = No

Q9 If yes, do you use any of the following classification systems?

- Mozena (1)
- Zuber (2)
- Own (3)
- Other- please specify (4) __________________________________________________

Q10 What treatment(s) do you offer? Please tick all that apply

- Nail cutting advice (1)
- Footwear/hygiene advice (2)
- Partial resection/removal of spicule (3)
- "Packing" (insertion of gauze/cotton wool etc to sulci) (4)
- Guttering (5)
- Nail brace (6)
- Nail avulsion without matrixectomy (7)
- Nail avulsion with matrixectomy (8)
- Other not listed- please specify (9) __________________________________________________

Q11 If you offer "packing" what material do you use?

- Sterile gauze/cotton wool/dressing (1)
- Non-sterile gauze/cotton wool/dressing (2)
- Other- please specify (4) __________________________________________________
- Do not offer (5)

Q12 If you use nail bracing, what system do you use?

- Specify below (1) __________________________________________________
- Do not offer (2)

End of Block: Treatment of ingrowing toenails

Start of Block: Nail Surgery

Q13 Do you perform nail surgery? (for the purposes of this survey, nail surgery refers to nail avulsion with/without chemical matrixectomy)

- Yes (1)
- No (2)

Skip To: Q14 If Do you perform nail surgery? (for the purposes of this survey, nail surgery refers to nail avulsi... = No

Skip To: Q15 If Do you perform nail surgery? (for the purposes of this survey, nail surgery refers to nail avulsi... = Yes

Q14 If no, what are the reasons for not performing nail surgery? (tick all that apply)

- Not part of current job role (2)
- Fear of litigation (3)
- Not enough demand (4)
- Work alone (5)
- Other- please specify (7) __________________________________________________

Skip To: End of Survey If Condition: If no, what are the reasons... Is Greater Than or Equal to 1. Skip To: End of Survey.

Q15 How many nail surgeries do you perform in an average month?

- <5 (1)
- 5-10 (2)
- 11-20 (3)
- 21+ (4)

Q16 Which of the following do you consider as indications for performing nail surgery? (please tick all that apply)

- Acute ingrowing nail(s) (1)
- Cosmesis (2)
- Failed conservative care (3)
- Fungal nail infection (4)
- Misshapen nails (5)
- Parental choice (6)
- Pincer nail (7)
- Psoriatic nail (8)
- Repeated ingrowing nail(s) (9)
- Other- please specify (10) __________________________________________________

Q17 If there is local infection present, do you request/provide a course of antibiotics prior to surgery?

- Yes (1)
- No (2)
- Sometimes (4)

Q18 Do you perform any other types of nail surgery? (tick all that apply)

- No (1)
- Suppan procedure (2)
- Zadiks procedure (3)
- Winograd procedure (4)
- Frost procedure (5)
- Other-pase specify (6) __________________________________________________

Q19 Do/have you ever referred to a surgeon for other types of surgery?

- Yes (1)
- No (2)
- I am a podiatric surgeon (3)

Skip To: Q21 If Do/have you ever referred to a surgeon for other types of surgery? = No

Skip To: Q20 If Do/have you ever referred to a surgeon for other types of surgery? = Yes

Q20 For what reasons did/would you refer? (tick all that apply)

- Medical considerations (1)
- Failed chemical matrixectomy (2)
- General anaesthetic preferred due to anxiety (3)
- Other- please specify (4) __________________________________________________

Q21 How would you manage patients with the following conditions?

|  | Go ahead as normal (1) | Go ahead without matrixectomy (2) | Refer to NHS podiatry (3) | Liaise with GP (4) | Liaise with consultant (5) | Refer for surgical consultation (6) | Delay surgery (7) | Would not perform surgery (8) | Unsure (9) |
| --- | --- | --- | --- | --- | --- | --- | --- | --- | --- |
| Diabetes (low risk) (1) |  |  |  |  |  |  |  |  |  |
| Diabetes (medium risk) (10) |  |  |  |  |  |  |  |  |  |
| Diabetes (high risk) (12) |  |  |  |  |  |  |  |  |  |
| Hepatic disease (2) |  |  |  |  |  |  |  |  |  |
| Auto-immune disorder (3) |  |  |  |  |  |  |  |  |  |
| HIV (4) |  |  |  |  |  |  |  |  |  |
| Endocarditis (5) |  |  |  |  |  |  |  |  |  |
| Autonomic Sympathetic Dysreflexia (6) |  |  |  |  |  |  |  |  |  |
| Kidney disease (7) |  |  |  |  |  |  |  |  |  |
| Pregnancy (8) |  |  |  |  |  |  |  |  |  |

Q22 How would you manage patients taking any of the following medications?

|  | Go ahead as normal (1) | Go ahead without matrixectomy (2) | Refer to NHS podiatry (3) | Liaise with GP (4) | Liaise with consultant (5) | Refer for surgical consultation (6) | Delay surgery (7) | Would not perform surgery (8) | Unsure (9) |
| --- | --- | --- | --- | --- | --- | --- | --- | --- | --- |
| Anticoagulant therapy (Warfarin, Aspirin etc) (1) |  |  |  |  |  |  |  |  |  |
| Cytokine Inhibitors (Adalimumab etc) (2) |  |  |  |  |  |  |  |  |  |
| Oral retinoids (3) |  |  |  |  |  |  |  |  |  |
| Antidepressants (4) |  |  |  |  |  |  |  |  |  |
| Antihypertensive medication (5) |  |  |  |  |  |  |  |  |  |
| Antiepileptic medication (6) |  |  |  |  |  |  |  |  |  |
| Anticonvulsant medication (7) |  |  |  |  |  |  |  |  |  |

End of Block: Nail Surgery

Start of Block: Pre-procedure

Q23 Do you use a consent form?

- Yes- own (1)
- Yes- provided by professional body (2)
- Yes- Local NHS trust (3)
- No (4)

Q24 Do you normally perform surgery alone, with another podiatrist or with an assistant? (tick all that apply)

- Alone (1)
- With another podiatrist (2)
- With assistant (3)
- Other- please specify (4) __________________________________________________

Q25 Does your place of work follow nail surgery guidelines?

- Yes- College of Podiatry guidelines (1)
- Yes- Other professional body (please specify) (2) __________________________________________________
- Yes- Local NHS guideline (3)
- Yes- Other (please specify) (4) __________________________________________________
- No (5)
- Unsure (6)

Q26 What vascular check(s) (if any) do you ROUTINELY perform prior to surgery (tick all that apply)

- None (1)
- Temp/colour assessment (2)
- Palpation of pulses (3)
- Check pulses with doppler (4)
- Capillary refill (5)
- Ankle-Brachial Pressure Index (ABPI) (6)
- Other- please specify (7) __________________________________________________

Q27  What neurological check(s) (if any) do you ROUTINELY perform prior to surgery? (tick all that apply)

- None (1)
- Monofilament (2)
- Tuning fork (3)
- Vibratip (4)
- Neuothesiometer (5)
- Other- please specify (6) __________________________________________________

End of Block: Pre-procedure

Start of Block: Procedure

Q28 Do you wear gloves during the procedure?

- Yes- sterile gloves (1)
- Yes- non-sterile gloves (2)
- No gloves (3)

Q29 Do you wear any of the following during the procedure?

|  | Yes (1) | No (2) |
| --- | --- | --- |
| Hair net (1) |  |  |
| Mask (2) |  |  |
| Apron (3) |  |  |
| Safety glasses (4) |  |  |

Q30 Which local anaesthetic(s) do you use? (tick all that apply)

- Lidocaine (1)
- Mepivacaine (2)
- Prilocaine (3)
- Bupivocaine (4)
- Levobupivocaine (5)
- Ropivocaine (6)
- Lidocaine with adrenaline (7)
- Bupivocaine with adrenaline (8)
- Other- please specify (9) __________________________________________________

Q31 What skin disinfectant do you usually use?

- Chlorhexidine (1)
- Clinisept (2)
- Hibiscrub (3)
- Iodine (4)
- Other- please specify (5) __________________________________________________
- None (6)

Q32 Do you use a tourniquet?

- Yes- tourniquet (1)
- Yes- tournicot (2)
- None (3)

Q33 Do you routinely include chemical matrixectomy when performing nail surgery?

- Yes (1)
- No (2)

Skip To: End of Block If Do you routinely include chemical matrixectomy when performing nail surgery? = No

Q34 Which chemical do you use for matrixectomy?

- Phenol (1)
- Sodium hydroxide (2)
- Trichloroacetic acid (3)
- Other- please specify (4) __________________________________________________

Display This Question:

If Which chemical do you use for matrixectomy? = Phenol

Q35 If phenol, do you use liquid phenol or EZ swabs?

- Liquid phenol (1)
- EZ swabs (2)
- Other- please specify (3) __________________________________________________

Q36 When thinking about applying the chemical, how many applications do you apply?

- 1 (1)
- 2 (2)
- 3 (3)
- 4 (4)
- More than 4- please specify (5) __________________________________________________

Q37 What is the TOTAL application time?

- 1 min (1)
- 2 min (2)
- 3 mins (3)
- 4 min (4)
- More than 4 min- please specify (5) __________________________________________________

Q38 Do you protect/mask the surrounding skin?

- Yes- please specify with what (1) __________________________________________________
- No (2)

Q39 Do you use a black file (or equivalent) to scarify the skin

- Yes (1)
- No (2)

Q40 If there is hypergranulation tissue present, do you use sharp dissection to remove?

- Yes- usually (1)
- Yes- sometimes (2)
- No (3)

Q41 What do you use to irrigate/flush the area?

- Chlorhexidine (1)
- Clinisept (2)
- Saline solution (3)
- Other- please specify (4) __________________________________________________
- None- do not irrigate (5)

Q42 For an average surgery without complications, what dressing(s) do you normally apply immediately after the procedure?

|  | Please specify (1) |
| --- | --- |
| Primary dressing (against wound) (1) |  |
| Secondary dressing (2) |  |
| Tertiary dressing materials (bandages/gauzes etc (3) |  |

End of Block: Procedure

Start of Block: Post-operative care

Q43 What products do you routinely provide for aftercare? (please tick all that apply)

- Nothing (1)
- Advice sheet (2)
- Dressings (3)
- Tubegauze (or equivalent) (4)
- Hypafix/tape (5)
- Saline solution (6)
- Clinisept (7)
- Other- please specify (8) __________________________________________________

Q44 How many follow up appointments do you usually provide for a TOTAL nail avulsion? (assuming no complications)

- None (1)
- 1-3 (2)
- 4-6 (3)
- 7-9 (4)
- 10+ (5)

Q45 How many follow up appointments do you usually provide for a PARTIAL nail avulsion? (assuming no complications)

- None (1)
- 1-3 (2)
- 4-6 (3)
- 7-9 (4)
- 10+ (5)

Q46 How long after surgery is your first follow up appointment? (if any)

- 1-3 days (1)
- 3-7 days (2)
- 7 days+ (3)
- Only see if complications (4)

Q47 How long would you expect healing to take place for a TOTAL nail avulsion (assuming no complications)

- 1-3 weeks (1)
- 4-6 weeks (2)
- 7-9 weeks (3)
- 10-12 weeks (4)
- 13-15 weeks (5)
- 16+ weeks (6)

Q48 How long would you expect healing to take place for a PARTIAL nail avulsion (assuming no complications)

- 1-3 weeks (1)
- 4-6 weeks (2)
- 7-9 weeks (3)
- 10-12 weeks (4)
- 13-15 weeks (5)
- 16+ weeks (6)

Q49 In your opinion what is the most common reason for delayed healing?

- Infection (1)
- Medical issues (2)
- Trauma (3)
- Non-adherence to postoperative care plan (4)
- Practitioner error (5)
- Other- please specify (6) __________________________________________________

Q50 Do you advise your patient to clean/irrigate the wound between dressing changes?

- No- Keep dry (1)
- Yes- Sterile saline solution (Irriclens, Stericlens etc) (2)
- Yes- Clinisept (3)
- Yes- Salt water bathing (4)
- Yes- Shower only (5)
- Yes- Bathe as normal (6)
- Yes- Other (please specify) (7) __________________________________________________

End of Block: Post-operative care

Start of Block: Reviewing clinical outcomes

Q51 Do you/your department evaluate the outcomes of patients receiving nail surgery in your clinic?

- Yes (1)
- No (2)

Skip To: End of Survey If Do you/your department evaluate the outcomes of patients receiving nail surgery in your clinic? = No

Q52 If yes, how long after the procedure? (tick all that apply)

- 1-6 months (1)
- 7-11 months (2)
- 1 year/1 year + (3)

Q53 How do you conduct the audit?

- Follow up appointment (1)
- Telephone (2)
- Postal (3)
- Other- please specify (4)

Q54 What outcomes do you collect, if any? (tick all that apply)

- None (1)
- Time to healing (2)
- Regrowth (3)
- Recurrence (4)
- Patient satisfaction (5)
- Other- please specify (6) __________________________________________________

Q55 If you use a professional standard/guideline, do you/your department audit your compliance with it?

- Yes (1)
- No (2)
- Unsure (3)

End of Block: Reviewing clinical outcomes

**Supplementary Figure 1**


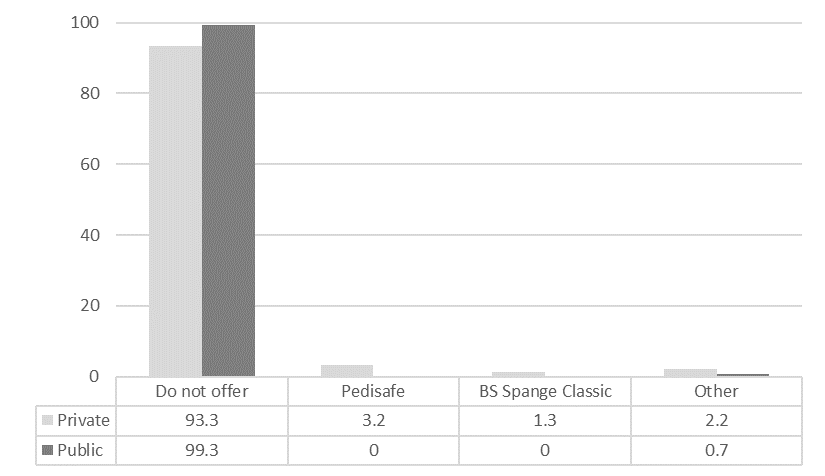

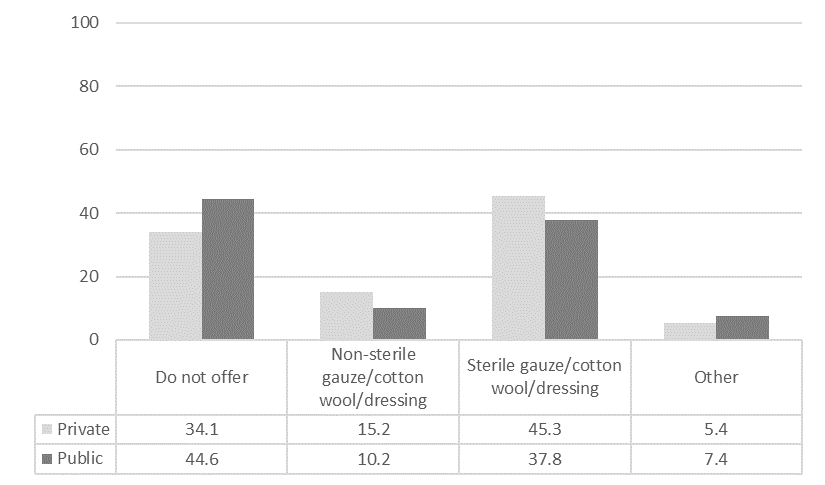


**(B)**

**(A)**

Private

Public


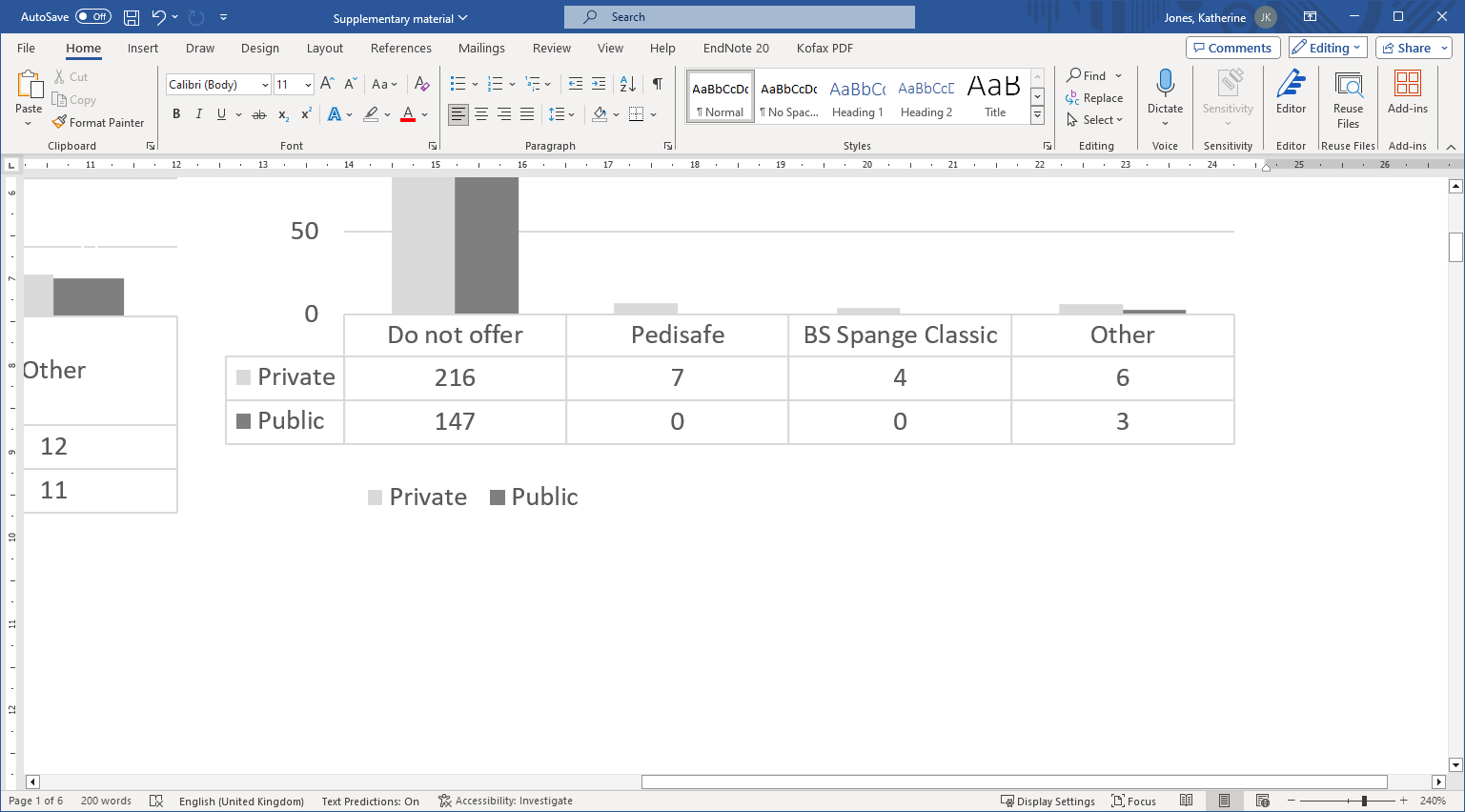


**Percentage of respondents (%)**

**Percentage of respondents (%)**

®

®

93.3% (n=210) 3.2% (n=7) 1.3% (n=3) 2.2% (n=5)

34.1% (n=76) 15.2% (n=34) 45.3% (n=101) 5.4% (n=12)

99.3% (n=147) 0% (n=0) 0% (n=0) 0.7% (n=1)

44.6% (n=66) 10.2% (n=15) 37.8% (n=56) 7.4% (n=11)

**‘Packing’ offered**

**Nail bracing offered**

**Supplementary Figure 1.** If and what type of ‘packing’ (A) (n=371) and nail bracing (B) (n=373) is offered

| **Supplementary Table 1.** Surgical Characteristics | | | |
| --- | --- | --- | --- |
|  | **Private Sector [n=188]** | **Public Sector [n=140]** | **All**  **[n=328]** |
| **Pre-procedure** | | | |
| **Consent form used, n (%)*^a^*** |  |  |  |
| Own consent form | 125 (79.1) | 6 (5.2) | 131 (47.8) |
| Consent form provided by professional body | 32 (20.3) | 2 (1.7) | 34 (12.4) |
| Consent from local NHS trust | 1 (0.6) | 106 (91.4) | 107 (39.1) |
| No consent form used | 0 (0.0) | 2 (1.7) | 2 (0.7) |
| **Antibiotics given if local infection present, n (%)** |  |  |  |
| Yes | 64 (34.0) | 41 (29.3) | 105 (32.0) |
| No | 22 (11.7) | 28 (20.0) | 50 (15.2) |
| Sometimes | 102 (54.3) | 71 (50.7) | 173 (52.8) |
| **Procedure** | | | |
| **Use of gloves, n (%)*^b^*** |  |  |  |
| Sterile gloves | 143 (92.3) | 107 (93.0) | 250 (92.6) |
| Non-sterile gloves | 12 (7.7) | 6 (5.2) | 18 (6.7) |
| No gloves | 0 (0.0) | 2 (1.8) | 2 (0.7) |
| **Use of a hair net, n (%) *^b^*** |  |  |  |
| Yes | 26 (16.8) | 22 (19.1) | 48 (16.0) |
| No | 129 (83.2) | 93 (80.9) | 227 (84.0) |
| **Use of a mask, n (%) *^b^*** |  |  |  |
| Yes | 111 (71.6) | 75 (65.2) | 186 (68.9) |
| No | 44 (28.4) | 40 (34.8) | 84 (31.1) |
| **Use of an apron, n (%) *^b^*** |  |  |  |
| Yes | 143 (92.3) | 109 (94.8) | 252 (93.3) |
| No | 12 (7.7) | 6 (5.2) | 18 (6.7) |
| **Use of safety glasses, n (%) *^b^*** |  |  |  |
| Yes | 78 (50.3) | 80 (69.6) | 158 (58.5) |
| No | 77 (49.7) | 35 (30.4) | 112 (41.5) |
| **Use a blacks file to scarify the skin, n (%) *^c^*** |  |  |  |
| Yes | 92 (67.6) | 62 (57.4) | 154 (63.1) |
| No | 44 (32.4) | 46 (42.6) | 90 (36.9) |
| **Chemical used for matrixectomy, n (%)*^d^*** |  |  |  |
| Phenol | 141 (99.3) | 106 (95.5) | 247 (97.2) |
| *Type of Phenol^e^:* Liquid Phenol | 86 (63.7) | 54 (51.9) | 140 (58.6) |
| EZ Swabs® | 43 (31.9) | 48 (46.2) | 91 (38.1) |
| Other | 6 (4.4) | 2 (1.9) | 8 (3.3) |
| Sodium Hydroxide | 0 (0.0) | 5 (4.5) | 5 (2.0) |
| Trichloroacetic acid | 1 (0.7) | 1 (0.9) | 2 (0.8) |
| **Aftercare, n (%) *^f^**** |  |  |  |
| Advice sheet | 144 (96.6) | 105 (94.6) | 249 (95.8) |
| Dressings | 138 (92.6) | 84 (75.7) | 222 (85.4) |
| Tubegauz (or equivalent) | 103 (69.1) | 39 (35.1) | 142 (54.6) |
| Hypafix®/tape | 101 (67.8) | 44 (39.6) | 145 (55.8) |
| Saline solution | 9 (6.0) | 1 (0.9) | 10 (3.8) |
| Clinisept® | 15 (10.1) | 0 (0.0) | 15 (5.8) |
| Other | 15 (10.1) | 9 (8.1) | 24 (9.2) |
| Nothing | 2 (1.3) | 3 (2.7) | 5 (1.9) |
| **Follow-up** | | | |
| **Expected healing time for total nail avulsion, n (%)*^f^*** |  |  |  |
| 1-3 weeks | 7 (4.7) | 2 (1.8) | 9 (3.5) |
| 4-6 weeks | 65 (43.6) | 40 (36.0) | 105 (40.4) |
| 7-9 weeks | 59 (39.6) | 44 (39.6) | 103 (39.6) |
| 10-12 weeks | 18 (12.1) | 25 (22.5) | 43 (16.5) |
| **Expected healing time for partial nail avulsion, n (%) *^f^*** |  |  |  |
| 1-3 weeks | 15 (10.1) | 7 (6.3) | 22 (8.5) |
| 4-6 weeks | 111 (74.5) | 82 (73.9) | 193 (74.2) |
| 7-9 weeks | 17 (11.4) | 19 (17.1) | 36 (13.8) |
| 10-12 weeks | 6 (4.0) | 3 (2.7) | 9 (3.5) |
| **Advice given to clean/irrigate the wound between dressing changes, n (%) *^f^**** |  |  |  |
| Keep dry | 24 (16.1) | 15 (13.5) | 39 (15.0) |
| Sterile saline solution | 15 (10.1) | 8 (7.2) | 23 (8.8) |
| Clinisept® | 12 (8.1) | 1 (0.9) | 13 (45.0) |
| Saltwater bathing | 93 (62.4) | 72 (64.9) | 165 (63.5) |
| Shower only | 11 (7.4) | 18 (16.2) | 29 (11.2) |
| Bathe as normal | 1 (0.7) | 7 (6.3) | 8 (3.1) |
| Other | 10 (6.7) | 5 (4.5) | 15 (5.8) |
| **Most common reason for delayed healing, n (%) *^f^*** |  |  |  |
| Infection | 33 (22.1) | 24 (21.6) | 57 (21.9) |
| Non-adherence to postoperative care plan | 81 (54.4) | 74 (66.7) | 155 (59.6) |
| Trauma | 16 (10.7) | 1 (0.9) | 17 (6.5) |
| Practitioner error | 2 (1.3) | 2 (1.8) | 4 (1.5) |
| Medical issues | 8 (5.4) | 3 (2.7) | 11 (4.2) |
| Other | 9 (6.0) | 7 (6.3) | 16 (6.2) |
| **Evaluate outcomes after nail surgery, n (%) *^f^*** |  |  |  |
| Yes | 71 (47.7) | 63 (56.8) | 134 (51.5) |
| No | 78 (52.3) | 48 (43.2) | 126 (48.5) |
| **If yes, outcomes collected, n (%)*^g^**** |  |  |  |
| None | 2 (3.0) | 0 (0.0) | 2 (1.6) |
| Time to healing | 37 (56.1) | 32 (54.2) | 69 (55.2) |
| Regrowth | 42 (63.6) | 45 (76.3) | 87 (69.6) |
| Recurrence | 25 (37.9) | 25 (42.4) | 50 (40.0) |
| Patient satisfaction | 61 (92.4) | 54 (91.5) | 115 (92.0) |
| Other | 3 (4.5) | 8 (13.6) | 11 (8.8) |
| **If yes, how long after procedure, n (%) *^g^*** |  |  |  |
| 1-6 months | 43 (65.2) | 31 (52.5) | 74 (59.2) |
| 7-11 months | 8 (12.1) | 8 (13.5) | 16 (12.8) |
| 1 year + | 15 (22.7) | 20 (34.0) | 35 (28.0) |
| **If yes, how are outcomes collected, n (%) *^g^*** |  |  |  |
| Follow-up appointment | 40 (60.6) | 12 (20.3) | 52 (41.6) |
| Telephone | 15 (22.7) | 26 (44.1) | 41 (32.8) |
| Postal | 0 (0.0) | 10 (16.9) | 10 (8.0) |
| Other | 11 (16.7) | 11 (18.7) | 22 (17.6) |
| **Use of a professional standard/guideline to audit compliance, n (%) *^g^*** |  |  |  |
| Yes | 20 (30.3) | 30 (50.8) | 50 (40.0) |
| No | 35 (53.0) | 13 (22.0) | 48 (38.4) |
| Unsure | 11 (16.7) | 16 (27.1) | 27 (21.6) |
| *^a^* 16.5% (n=54/328) respondents did not complete this question  *^b^* 17.7% (n=58/328) respondents did not complete this question  *^c^* 25.6% (n=84/328) respondents did not complete this question  *^d^* 25.6 (n=74/328) respondents did not complete this question  *^e^* 3.2% (n=8/247) respondents did not complete this question  *^f^* 20.7% (n=68/328) respondents did not complete this question  ***^g^*** 6.7% (n=9/134) respondents did not complete this question  *Multiple answers possible | | | |

**Supplementary Figure 2**

**Number of respondents**


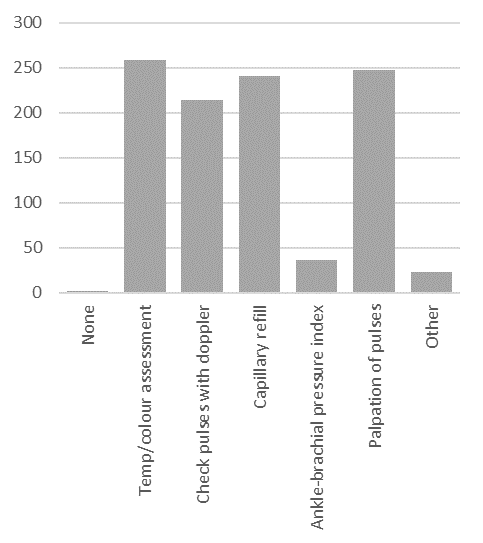


**(B)**

**Vascular checks**

**Neurological checks**

**Number of respondents**

**Supplementary Figure 2.** What vascular checks (A) and neurological checks (B) are performed routinely prior to surgery (n=274*)

**Multiple answers possible*

**(A)**


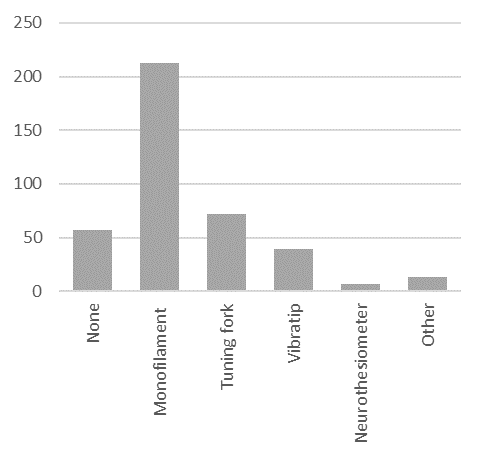


| **Supplementary Table 2**. Management of patients with certain health conditions and taking certain medication (n=275) | | | | | | | | | |
| --- | --- | --- | --- | --- | --- | --- | --- | --- | --- |
| **Management of patients with certain health conditions (n=275)** | | | | | | | | | |
| Condition | Go ahead as normal | Go ahead without matrixectomy | Liase with consultant | Liase with GP | Refer to surgeon | Refer to NHS podiatry | Delay surgery | Not perform surgery | Unsure |
| Diabetes (Low risk) | 224 (81.5%) | 4 (1.5%) | 0 (0%) | 38 (13.8%) | 0 (0%) | 9 (3.3%) | 0 (0%) | 0 (0%) | 0 (0%) |
| Diabetes (Medium risk) | 113 (41.1%) | 7 (2.5%) | 10 (3.6%) | 101 (36.7%) | 0 (0%) | 33 (12.0%) | 5 (1.8%) | 4 (1.5%) | 2 (0.7%) |
| Diabetes (High risk) | 29 (10.5%) | 11 (4.0%) | 36 (13.1%) | 62 (22.5%) | 11 (4.0%) | 83 (30.2%) | 8 (2.9%) | 32 (11.6%) | 3 (1.1%) |
| Hepatic disease | 32 (11.6%) | 2 (0.7%) | 61 (22.2%) | 102 (37.1%) | 9 (3.3%) | 46 (16.7%) | 1 (0.4%) | 14 (5.1%) | 8 (2.9%) |
| Auto-immune disorder | 32 (11.6%) | 5 (1.8%) | 71 (25.8%) | 103 (37.5%) | 5 (1.8%) | 42 (15.3%) | 3 (1.1%) | 8 (2.9%) | 6 (2.2%) |
| HIV | 59 (21.5%) | 3 (1.1%) | 50 (18.2%) | 64 (23.2%) | 10 (3.6%) | 57 (20.7%) | 0 (0%) | 16 (5.8%) | 16 (5.8%) |
| Endocarditis | 30 (10.9%) | 2 (0.7%) | 60 (21.8%) | 88 (32.0%) | 6 (2.2%) | 44 (16.0%) | 2 (0.7%) | 28 (10.2%) | 15 (5.5%) |
| Autonomic sympathetic dysreflexia | 13 (4.7%) | 1 (0.4%) | 53 (19.3%) | 57 (20.7%) | 26 (9.5%) | 49 (17.8%) | 1 (0.4%) | 30 (10.9%) | 45 (16.4%) |
| Kidney disease | 27 (9.8%) | 3 (1.1%) | 68 (24.7%) | 104 (37.8%) | 9 (3.3%) | 44 (16.0%) | 1 (0.4%) | 17 (6.2%) | 2 (0.7%) |
| Pregnancy | 12 (4.4%) | 16 (5.8%) | 2 (0.7%) | 32 (11.6%) | 1 (0.4%) | 12 (4.4%) | 142 (51.6) | 49 (17.8%) | 9 (3.3%) |
| **Management of patients taking certain medication (n=275)** | | | | | | | | | |
| Anticoagulant therapy | 64 (23.2%) | 1 (0.4%) | 19 (6.9%) | 152 (55.3%) | 5 (1.8%) | 25 (9.1%) | 4 (1.5%) | 2 (0.7%) | 1 (0.4%) |
| Cytokine inhibitors | 22 (8.0%) | 3 (1.1%) | 72 (26.2%) | 93 (33.8%) | 10 (3.6%) | 35 (12.7%) | 2 (0.7%) | 6 (2.2%) | 32 (11.6%) |
| Oral retinoids | 43 (15.6%) | 3 (1.1%) | 28 (10.2%) | 109 (39.6%) | 5 (1.8%) | 19 (6.9%) | 14 (5.1%) | 11 (4.0%) | 43 (15.6%) |
| Antidepressants | 172 (62.5%) | 3 (1.1%) | 2 (0.7%) | 80 (29.1%) | 1 (0.4%) | 4 (1.5%) | 4 (1.5%) | 2 (0.7%) | 7 (2.5%) |
| Antihypertensive medication | 205 (74.5%) | 3 (1.1%) | 0 (0%) | 58 (21.1%) | 2 (0.7%) | 7 (2.5%) | 0 (0%) | 0 (0%) | 0 (0%) |
| Antiepileptic medication | 92 (33.5%) | 1 (0.4%) | 14 (5.1%) | 120 (43.6%) | 5 (1.8%) | 27 (9.8%) | 0 (0%) | 6 (2.2%) | 10 (3.6%) |
| Anticonvulsant medication | 71 (25.8%) | 1 (0.4%) | 23 (8.4%) | 121 (44.0%) | 8 (2.9%) | 29 (10.5%) | 0 (0%) | 9 (3.3%) | 13 (4.7%) |
| n, Number; HIV, Human Immunodeficiency Virus; GP, General Practitioner | | | | | | | | | |

**Supplementary Figure 3**

Private

Public

**Disinfectant used**


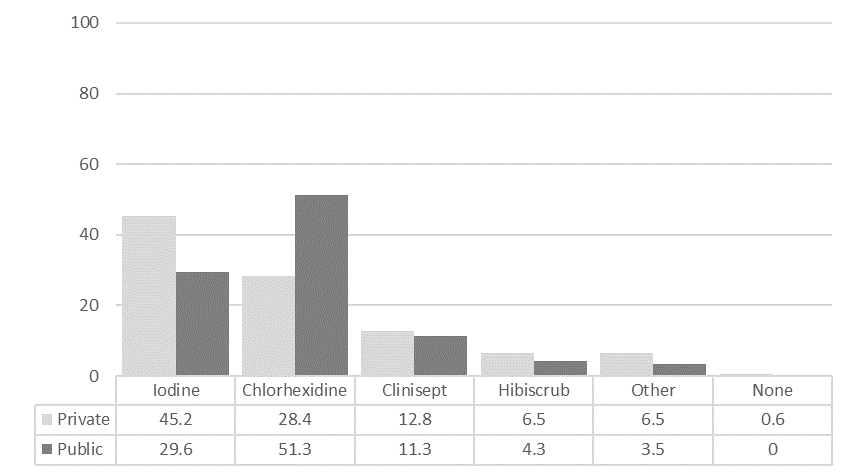


45.2% (n=70) 28.4% (n=44) 12.8% (n=20) 6.5% (n=10) 6.5% (n=10) 0.6% (n=1)

29.6% (n=34) 51.3% (n=59) 11.3% (n=13) 4.3% (n=5) 3.5% (n=4) 0.0% (n=0)

**Percentage of respondents (%)**

®

®

**Supplementary Figure 3.** Disinfectant used in the private (n=155) and public sector (n=115)

| **Supplementary Table 3.** Dressings applied immediately after procedure (without complications) | | | | | | |
| --- | --- | --- | --- | --- | --- | --- |
|  | **Primary** | | **Secondary** | | **Tertiary** | |
| **Dressings, n (%)*** | **Private [n= 136]** | **Public [n= 108]** | **Private [n= 136]** | **Public [n= 108]** | **Private [n= 136]** | **Public [n= 108]** |
| Adaptic touch® |  | 3 (2.8) |  |  |  |  |
| Aeropad® |  | 2 (1.9) |  | 5 (4.6) |  |  |
| Algeosteril® |  | 2 (1.9) |  | 2 (1.9) |  |  |
| Alginate (including calcium) |  | 1 (0.9) |  | 1 (0.9) |  |  |
| Allevyn® |  | 1 (0.9) | 2 (1.5) |  |  |  |
| Atrauman® | 5 (3.7) | 38 (35.2) | 1 (0.7) |  |  |  |
| Bactigras® | 38 (27.9) | 15 (13.9) |  |  |  |  |
| Betadine® | 2 (1.5) |  |  |  |  |  |
| Cutipast® |  |  | 1 (0.7) |  |  |  |
| Foam |  | 1 (0.9) |  | 3 (2.8) |  | 1 (0.9) |
| Gauze/ sterile gauze/ non adherent gauze | 4 (2.9) | 5 (4.6) | 37 (27.2) | 41 (38.0) | 72 (52.9) | 88 (81.5) |
| Haemostat | 1 (0.7) | 2 (1.9) |  |  |  |  |
| Hyperflix® or Mefix® |  |  |  |  | 34 (25.0) | 18 (16.7) |
| Inadine® | 28 (20.6) | 6 (5.6) | 1 (0.7) |  |  |  |
| Jelonet® | 19 (14.0) | 16 (14.8) |  |  |  |  |
| Kaltostat® | 21 (15.4) | 7 (6.5) | 20 (14.7) | 11 (10.2) |  |  |
| Melolin® | 17 (12.5) | 6 (5.6) | 70 (51.5) | 23 (21.3) | 10 (7.4) | 4 (3.7) |
| Melolite® | 1 (1.4) | 1 (0.9) |  | 5 (4.6) |  | 3 (2.8) |
| Mepitel® | 1 (0.7) | 3 (2.8) |  |  |  |  |
| Mepore® |  | 1 (0.9) |  | 1 (0.9) | 3 (2.2) |  |
| Podlin® |  |  | 4 (2.9) |  |  |  |
| PremierPore® |  |  | 1 (0.7) | 1 (0.9) |  |  |
| Relipad® | 1 (0.7) |  |  |  |  |  |
| Self-adherent bandage |  |  |  |  | 1 (0.7) | 4 (3.7) |
| Sorbsan® | 1 (0.7) |  |  | 4 (3.7) |  |  |
| Telfa® | 1 (0.7) | 3 (2.8) | 1 (0.7) | 10 (9.3) |  | 1 (0.9) |
| Tubular bandage |  |  |  | 2 (1.9) | 112 (82.4) | 75 (69.4) |
| N-A Ultra® | 3 (2.2) | 2 (1.9) |  |  |  |  |
| Urgo border® |  | 2 (1.9) |  |  |  |  |
| Nothing | 1 (0.7) | 1 (0.9) | 1 (0.7) | 3 (2.8) |  | 2 (1.9) |
| n, Number  **Multiple answers possible* | | | | | | |
